# Event and Comment

**Published:** 1905-01-15

**Authors:** 


### DENTAL REGISTER.

Vol. LIX. January 15, 1905. No. 1.

### EVENT AND COMMENT.

### Nineteen Hundred and Five.

With this issue the DENTAL REGISTER begins its fifty-ninth year of life. This is the longest continuous life any dental journal has as yet experienced. We old folks like to brag about our age, and would like to exercise our prerogative of reminiscing at length; but we have learned that the present age cares not so much for the past as for the present and the prospective, and we are therefore reluctantly compelled to suffer in silence and square away for action. What we have done the past year is in print. For some of it we ought to apologize, for much of it we are indebted to our kind friends and co-workers in the journalistic world. We have presented that which has come to us from first hands to the best of our ability and trust it has met the approval of our readers. What we have borrowed from our contemporaries we have selected with the best judgment we possess and cannot therefore apologize for that. Our printers have made some typographical, and we have made more grammatical and rhetorical errors, but we are trying to be more careful. Our publishers are kind and indulgent and we commend their interests most cordially to our old and the many new readers we expect to gain this year.

For the ensuing year we shall continue our several departments as at present, with the exception that we shall drop the Editorial department, as such, and substitute for it a department under the heading of "EVENT AND COMMENT" which will be transferred to the first and such succeeding pages as may be required. It will be our purpose to present in editorial manner such prominent events as may seem to us of sufficient interest to warrant special comment. We invite the co-operation of our readers in this venture, and promise to present any subject you may suggest to the best of our ability. We hope to receive from our readers and friends topics and comments which shall make this a valuable feature. That our readers may enjoy a prosperous and happy New Year is our most cordial greeting.

---

**Germany and the American Degree.**

By several communications published in the January *Items of Interest,* it seems that there is a crusade against the holders of the American Dental Degree in Germany. The Imperial Court of Germany has ruled that only the degrees of dental departments of State controlled universities will be tolerated in that country. It seems therefore that the status of other American degree holders is little or no better than the so-called "Technicker," who is merely a laboratory man, and never attempts any surgical work. This ruling, of course, is causing much anxiety among American degree holders, many of whom are Germans who expended much time and money to secure the American qualification. To have to be put on an equality with the laboratory man, and deprived of legal sanction in using any sort of medicine is certainly a great wrong. The aggrieved parties are now appealing to the United States diplomatic department for interference on their behalf. In strict accordance with this ruling only the degrees of such institutions as the University of Michigan, Minnesota and Iowa can be recognized. As these are the only State institutions wholly endowed and controlled by the States. Comparatively few of the diploma holders in Germany are graduates of these institutions.

---

**Tri-State Meeting.**

The series of meetings which have been held under the auspices of the Ohio, Indiana and Michigan State Societies, the last of which was held at Indianapolis three years ago, seems destined to be discontinued, at least for the present. Each of the States has acted the part of entertainer, and the Michigan Society has again invited the Ohio and Indiana Societies to meet with it the coming summer at Detroit. The Ohio State Society at its recent meeting declined the invitation, and the tri-state meeting will therefore be declared off, for the present at least, as there is not sufficient time to organize the meeting with other societies which might wish to attend, as they already have their arrangements for their regular meetings well under way. The reason given by the Ohio Society for declining is that this society meets in regular session in December and can not therefore drop a meeting, as the period between its sessions would be so long as to invite disaster to future attendance by breaking into the established habit of its members of meeting once each year in December. The necessity also of preparing two programs for the year in which the tri-state meeting is held makes too much of a burden for the members and officers. Another reason given is that the conviction seems to be that the members of the State Societies get more benefit from attending their own meetings where they know every one and are known, than in the large gatherings where the essayists are unknown or perhaps speak in unknown tongues on impracticable topics of little value or interest to the average practitioner; and the clinicians and exhibits are so thronged that little can be gained from a bird's eye view, which is many times all one can get. All these objections are perhaps true and have force, and yet it does not seem that they ought to apply in this instance. These meetings have not perhaps been great technical or scientific successes, yet they have accomplished much along these lines that is well well worth while. We don't believe any state meeting was ever held in either of the three states that could compare in value with the work done and real professional good accomplished by any of the three tri-state meetings. The programs in every case were excellent, and the clinics were many and of great variety; and no better exhibit of supplies was ever made than at the Indianapolis meeting. But the one great feature, which ought to be perpetuated, is the spontaneous development of good cheer and professional fellowship which has characterized every one of these meetings. We hope they shall not be permanently dropped.

---

**Institute of Dental Pedagogics.**

The twelfth annual meeting of this society was held at Louisville, December 28th, 29th and 30th, at the invitation of the Louisville College of Dentistry and the Falls City Dental Club. The attendance was not as large as usual, but the interest in the meeting was characteristic of this society. There seems to be a relaxation in all society work this year probably because of the great effort made to carry out the plans of the International Congress at St. Louis the past summer. The program of the institute this year went farther away from the technical subjects than ever before. The original organizers of this society had little idea that such subjects should find their way into the programs for discussion as two of the numbers on the program this year. "Faces of Pluggers," and "A Pharmacological Study of Silver Nitrate," cannot in themselves offer much of pedagogic value, and yet as examples of methods of investigation and for class presentation even such apparently irrelevant subjects have very great value. The interest in the meeting was good, and those who attended say it was one of the best meetings of the year. The peculiar fact about these meetings is that practitioners who attend them invariably speak enthusiastically of them. The dentists of Louisville lived up to their traditional standard of hospitality, and the meetting was a success socially as well as educationally. The meeting will be held next year in New York City. It is hoped that the Eastern men may attend more generously than heretofore.

---

**Illinois State Re-organization.**

The Illinois State Dental Society has undertaken to systematically organize the profession into co-operating local or district societies. The plan is to organize by counties or favorably-located districts, and each of these local societies is to be affiliated with the State society. To become a member of the State society, one must join his local society first, as he cannot join the State society in any other way. The dues of the local society will be so much per year as each local society may of its own choice adopt, but the local society must contribute two dollars per member each year to the State society. This entitles the members of the local society to all the privileges of the State society and its published transactions, and any other benefits which may accrue from such a membership. It is expected by this means to greatly augment the membership of the State society, in fact to secure the membership of practically every ethical practitioner in the State. The proceedings of all the societies will be carefully edited and published. By this plan it is hoped to bring more men into society work, and therefore greatly increase the intelligence of the profession. It certainly is a most attractive scheme, and we shall watch its development with interest.

### Emperor William's Dentist Suicides.

Dr. A. H. Sylvester, who has had the court patronage at Berlin for many years, and who enjoyed intimate friendly relations with the Emperor, committed suicide January 10th, by shooting himself through the head in his bed chamber. He had a severe attack of influenza which caused great suffering. For two or three days previous to his death he conversed incoherently. Dr. Sylvester graduated from the Boston Dental College in 1871, and located in Berlin soon afterward. He was a well-educated man and a skillful dentist, but never interested himself in public professional work, he is not known in either the technical, scientific or literary professional circles. He was however well-known to American travelers who had occasion to meet him socially in Berlin. He gave himself up to the social life in which he lived, and moved as a prince of good fellows. It is said of him that he made more money than any other American practicing in Europe, but he spent his money as freely as he made it, and consequently was frequently financially embarrassed. He had a checkered career in his domestic affairs, and in fact led his kind of life to the limit. He enjoyed it while it lasted, and it may be that he lived a long life so far as experience goes in the thirty years he has been in Germany. The question naturally arises, did he make a wise choice? Few would have the courage to begin such a career even should the opportunity offer.

---

### Quinine for Pyorrhoea.

Dr. Joseph Head, in the December *Items*, makes the following statement: After thoroughly cleansing the roots of teeth affected with pyorrhea, firmly fix the teeth with ligatures or splints and pack the pockets full with quinine sulphate as an antiseptic dressing. The quinine will exclude bacteria and cause the gums to become reattached to the peridental membrane or tooth substance. This is a rather strong statement and one that the pathology of this disease and the pharmacology of the drug will scarcely justify on logical grounds; but Dr. Head quotes Dr. James Truman as the author of the remedy and certifies it from his own clinical experience. We sometimes meet with unexpected therapeutic paradoxes in the clinical use of drugs, which lead us to lose confidence in scientific findings. A notable example is seen in iodoform, which Dr. Black found in his scientific research to have a very low value as an antiseptic, but which has been more used for this purpose by clinicians than any other drug, in fact the iodine fad promises to have but just begun to make itself felt. But the comment that we want to make on this treatment is from an altogether different view point. Quinine may be an ideal remedy for pyorrhea; but how many patients will submit to such a treatment! The taste of this drug is exceedingly distasteful to almost every one, even when taken in regular form and dosage for systemic impressions. We do not think many patients will be willing to have their gums impregnated with this bitter drug to be gradually dissolved in the mouth fluids and so manifest its bitterness for several hours or perhaps days. The remedy will be worse than the disease, and the dentist who makes use of it ought to be very sure of the loyalty of his patient. Dentists have not as yet reached the sugar-coated stage in therapeutics, and it is possible that their peculiar field is not adapted to that system; but there is too much of the old style use of radical remedies, or it may be thoughtless use of powerful or distasteful drugs. Irritants and cauterants as well as some of the most powerful poisons are used with an abandon about the mouth that no other structures of the body would tolerate. We felt justified recently in abandoning the use of a very effective disinfectant remedy because several patients remarked that it had a horrid taste.

**Dealers' Exhibit Convention.**

The enterprising firm, The Ransom & Randolph Co., of Toledo, held a three days' exhibit by manufacturers of dental supplies at their commodious sales-rooms in Toledo, December 12th to 14th. Almost every well-known manufacturer was represented, and the display was supplemented by clinics with appliances by men who were the original inventors, or were particularly skillful in using the appliances. The object of the convention was to show the goods in a comparative display. When it is stated that nearly three hundred dentists registered their attendance, and some of these came more than two hundred miles, we get some idea as to what some dentists would like to see at their conventions. Program committees take notice.

